# Relationship between LILRB2 and APE1 levels and pathological characteristics in colorectal cancer patients and their predictive value for prognosis

**DOI:** 10.3389/fcimb.2025.1630604

**Published:** 2025-11-26

**Authors:** Ji Li, Xiaofang Tang, MianYong Wu, JinMing Tu

**Affiliations:** 1 Gastroenterology Department, Longyou County People’s Hospital, Longyou, Quzhou, Zhejiang, China; 2 Gastroenterology Department, Tongxiang First People’s Hospital, Tongxiang, Jiaxing, Zhejiang, China

**Keywords:** colorectal cancer, Leukocyte Immunoglobulin-Like Receptor Subfamily B2 (LILRB2), Apurinic/apyrimidinic endonuclease 1 (APE1), pathological characteristics, prognosis, Cox regression analysis

## Abstract

**Objective:**

This study aimed to investigate the relationship between the expression levels of Leukocyte Immunoglobulin-Like Receptor Subfamily B2 (LILRB2) and Apurinic/Apyrimidinic Endonuclease 1 (APE1) and the pathological characteristics in colorectal cancer (CRC) patients, as well as their predictive value for prognosis.

**Methods:**

Serum levels of LILRB2 and APE1 were analyzed in CRC patients with varying pathological characteristics. The associations between LILRB2 and APE1 serum levels and patient prognosis was evaluated, and factors influencing prognosis were identified.

**Results:**

Patients with high LILRB2 expression exhibited a significantly lower survival rate than those with low expression, and the same trend was observed for APE1 expression (both *P* < 0.001). Univariate Cox analysis revealed that expression levels of LILRB2 and APE1, TNM stage, and lymph node metastasis (LNM) were associated with CRC prognosis. Multivariate Cox analysis demonstrated that high expression levels of LILRB2 and APE1, advanced TNM stage, and the presence of LNM were independent risk factors impacting CRC prognosis (*P* < 0.05).

**Conclusion:**

Abnormal expression levels of LILRB2 and APE1 are associated with age, tumor diameter, TNM stage, and LNM in CRC patients. Elevated expression of these markers predicts a poorer prognosis.

## Introduction

Colorectal cancer (CRC) ranks as the second most frequently diagnosed malignancy in adult women and the third in adult men, standing as the fourth leading cause of cancer-related mortality and accounting for approximately 9.2% of all cancer deaths globally (Bray et al., 2018; Dekker et al., 2019). Fewer than half of CRC cases are diagnosed at a locally advanced stage. The disease is highly heterogeneous and involves numerous genetic or somatic mutations (Zygulska and Pierzchalski, 2022). Its onset, progression, and prognosis are closely associated with the immune status of the tumor microenvironment (TME) and the metabolic activity of tumor cells (Tomasini et al., 2021; Ferkel et al., 2025; Liu et al., 2025). In recent years, increasing attention has been directed toward the roles of immune checkpoint molecules and DNA damage repair proteins in tumor progression.

Clinico-pathological characteristics such as tumor stage are well-established prognostic indicators for predicting patient survival (Brenner et al., 2014; Renfro et al., 2014). Leukocyte Immunoglobulin-Like Receptor B2 (LILRB2) is a transmembrane glycoprotein structurally similar to the immune checkpoint protein programmed cell death protein-1 (PD-1) and its ligand PD-L1. It is widely expressed on dendritic cells, macrophages, and other myeloid cells, where it inhibits immune cell activity and participates in important pathological processes, including modulation of the TME, immune evasion, and tumor progression (Chen et al., 2018). According to previous research, LILRB2 contributes to immune suppression and tumor advancement in malignancies such as acute myeloid leukemia and non-small cell lung cancer (Zhao et al., 2024). Moreover, LILRB2 expression is markedly upregulated in CRC, where its elevation correlates with unfavorable clinicopathological parameters, including poor-to-moderate differentiation, lymph node metastasis (LNM), advanced TNM stage, and poor prognosis (Wang et al., 2023). Analyses of T-cell subsets in CRC and lung adenocarcinoma have further revealed that LILRB2 overexpression is associated with decreased CD3+ and CD8+ T-cell levels and increased FOXP3+ regulatory T-cell infiltration within the TME (Cao et al., 2025).

Apurinic/apyrimidinic endonuclease 1 (APE1), a key enzyme in the base excision repair pathway, also functions as a redox-sensitive modulator that activates multiple transcription factors (Oliveira et al., 2022; Xue and Demple, 2022; Zhang et al., 2022). APE1 has been implicated in CRC progression, influencing tumor growth, chemotherapeutic resistance, and metastatic potential in both human CRC cells and carcinogen-induced CRC models. Elevated APE1 expression is closely linked to advanced disease stage and reduced survival, suggesting its potential as a prognostic and predictive biomarker for CRC (Hong et al., 2023).

Although LILRB2 and APE1 have been independently identified as regulators of tumor immunity and DNA repair, respectively, potential crosstalk or synergistic regulation between them in CRC remains unclear. Considering their associations with tumor pathology and patient prognosis, both molecules represent promising biomarkers. This study, therefore, integrates LILRB2 and APE1 within a unified analytical framework to investigate their co-expression patterns in CRC, their relationships with clinicopathological characteristics, and their combined predictive value for patient prognosis.

## Materials and methods

### Ethical approval

This study was approved by the Ethics Committee of Tongxiang First People’s Hospital. Written informed consent was obtained from all participants prior to enrollment.

### Study participants

From May 2019 to May 2021, 100 CRC patients treated at Tongxiang First People’s Hospital were enrolled as the study group. The inclusion criteria were as follows. (1) Meeting the diagnostic criteria for CRC (Cubiella et al., 2018); (2) Aged 18 to 80 years; (3) Primary surgical treatment without prior radiotherapy, chemotherapy, or anti-inflammatory therapy; (4) Availability of complete clinical and pathological data; (5) An expected survival time of more than 3 months. Exclusion criteria included: (1) Coexistence of another primary or metastatic malignant tumor; (2) Presence of systemic immune or hematological diseases; (3) Other colorectal lesions such as intestinal tuberculosis or colonic vascular diseases; (4) History of previous colorectal surgery; (5) Poor compliance or loss to follow-up. In addition, 80 healthy volunteers undergoing routine physical examinations during the same period were recruited as the control group. The control group consisted of 49 males and 31 females, aged 43 to 77 years, with a mean age of 59.23 ± 8.39 years. The study group consisted of 51 males and 49 females, aged 44 to 77 years, with a mean age of 60.06 ± 7.66 years. No marked differences were noted in gender or age between the two groups (*P* > 0.05), indicating comparability.

### Observational indicators

#### Collection of pathological characteristics

Clinicopathological parameters including age, gender, tumor location, tumor diameter, TNM stage, degree of differentiation, LNM, and neural invasion were collected from all CRC patients.

### Detection of serum LILRB2 and APE1 expression

Three milliliters of fasting venous blood were collected from each participant in the morning. After standing at ambient temperature for 1 hour, the samples were centrifuged at 3000 rpm/min for 10 minutes. The supernatant was collected and stored at -80 °C until analysis. Serum LILRB2 and APE1 levels were determined using enzyme-linked immunosorbent assay (ELISA) according to the manufacturer’s instructions.

### Prognosis assessment

Patients were followed up for two years via outpatient visits, telephone calls, or online communication at 6-month intervals. The follow-up period concluded in June 2023, and no cases were lost to follow-up. Overall survival was calculated from the completion of treatment to the last follow-up or the date of death.

### Statistical analysis

All statistical analyses were conducted using SPSS version 21.0 (IBM Corp., Armonk, NY, USA). Continuous variables were expressed as the mean ± standard deviation ( ± s) and compared between groups using the *t*-test. Categorical variables were presented as percentages (%) and analyzed using the χ² test. Kaplan-Meier survival curves were utilized to evaluate the relationship between serum expression levels of LILRB2 and APE1 and the prognosis of CRC patients. Univariate and multivariate Cox proportional hazards regression analyses were applied to identify independent prognostic factors. Receiver operating characteristic (ROC) curve analysis was used to evaluate the prognostic predictive value of LILRB2 and APE1 levels in CRC patients. A *P*-value of less than 0.05 was considered statistically significant.

## Results

### Expression levels of LILRB2 and APE1

The study group had significantly higher serum levels of LILRB2 and APE1 versus the control group (*P* < 0.05). These results indicate that CRC patients exhibited markedly elevated serum LILRB2 and APE1 levels compared with healthy controls (**Table 1**).

**Table 1:** Comparison of expression levels of LILRB2 and APE1 between the two groups.

Indicator	Control group (n = 80)	Study group (n = 100)	*t*	*P*
LILRB2 (pg/mL)	214.78 ± 46.58	402.18 ± 89.20	17.021	<0.001
APE1 (ng/mL)	0.13 ± 0.07	0.48 ± 0.11	24.214	<0.001

### Relationship between LILRB2 and APE1 serum levels and pathological characteristics in CRC patients

Based on the mean serum expression levels of LILRB2 and APE1 (LILRB2: 402.18; APE1: 0.48), the 100 CRC patients were stratified into high- and low-expression groups for each marker (LILRB2: n = 51 high, n = 49 low; APE1: n = 50 high, n = 50 low). Analysis revealed that LILRB2 expression differed significantly among patients of different ages, TNM stages, and LNM status (*P* < 0.05). However, LILRB2 levels were not significantly associated with gender, tumor location, tumor diameter, degree of differentiation, or neural invasion (*P* > 0.05). Similarly, APE1 expression levels showed significant differences according to tumor diameter, TNM stage, and LNM status (*P* < 0.05), but no significant correlations were found with age, gender, tumor location, degree of differentiation, or neural invasion (*P* > 0.05). These findings suggest that serum LILRB2 and APE1 expression levels are closely associated with age, tumor diameter, TNM stage, and LNM in CRC patients (**Table 2**).

**Table 2:** Relationship between serum expression levels of LILRB2 and APE1 and pathological characteristics in CRC patients.

Pathological characteristics	Number of cases	Low LILRB2 expression group (n = 49)	High LILRB2 expression group (n = 51)	*χ^2^*	*P*	Low APE1 expression group (n = 50)	High APE1 expression group (n = 50)	*χ^2^*	*P*
Age (years)				3.968	0.046			3.252	0.071
≤60	47	28 (57.14%)	19 (37.25%)			28 (56.00%)	19 (38.00%)		
>60	53	21 (42.86%)	32 (62.75%)			22 (44.00%)	31 (62.00%)		
Gender				2.575	0.109			3.241	0.072
Male	51	29 (59.18%)	22 (43.14%)			30 (60.00%)	21 (42.00%)		
Female	49	20 (40.82%)	29 (56.86%)			20 (40.00%)	29 (58.00%)		
Lesion location				2.019	0.155			0.644	0.422
Colon	46	19 (38.78%)	27 (52.94%)			21 (42.00%)	25 (50.00%)		
Rectum	54	30 (61.22%)	24 (47.06%)			29 (58.00%)	25 (50.00%)		
Tumor diameter (cm)				1.164	0.281			4.574	0.032
≤ 5	77	40 (81.63%)	37 (72.55%)			43 (86.00%)	34 (68.00%)		
> 5	23	9 (18.37%)	14 (27.45%)			7 (14.00%)	16 (32.00%)		
TNM stage				6.732	0.009			5.769	0.016
Stage I and II	48	30 (61.22%)	18 (35.29%)			30 (60.00%)	18 (36.00%)		
Stage III and IV	52	19 (38.78%)	33 (64.71%)			20 (40.00%)	32 (64.00%)		
Degree of differentiation				0.293	0.588			0.379	0.538
Poorly differentiated	12	5 (10.20%)	7 (13.73%)			7 (14.00%)	5 (10.00%)		
Moderately and well differentiated	88	44 (89.80%)	44 (86.27%)			43 (86.00%)	45 (90.00%)		
Lymph node metastasis				5.713	0.017			8.734	0.003
Yes	34	11 (22.45%)	23 (45.10%)			10 (20.00%)	24 (48.00%)		
No	66	38 (77.55%)	28 (54.90%)			40 (80.00%)	26 (52.00%)		
Perineural invasion				0.962	0.327			<0.001	1.000
Yes	42	23 (46.94%)	19 (37.25%)			21 (42.00%)	21 (42.00%)		
No	58	26 (53.06%)	32 (62.75%)			29 (58.00%)	29 (58.00%)		
Postoperative adjuvant chemotherapy				0.600	0.439			0.043	0.836
Yes	37	20 (40.82)	17 (33.33)			19 (38.00)	18 (36.00)		
No	63	29 (59.18)	34 (66.67)			31 (62.00)	32 (64.00)		
Hypertension				0.739	0.390			0.233	0.629
Yes	22	9 (18.37)	13 (25.49)			12 (24.00)	10 (20.00)		
No	78	40 (81.63)	38 (74.51)			38 (76.00)	40 (80.00)		
Diabetes				0.010	0.920			2.250	0.134
Yes	20	10 (20.41)	10 (19.61)			13 (26.00)	7 (14.00)		
No	80	39 (79.59)	41 (80.39)			37 (74.00)	43 (86.00)		

### Relationship between serum expression levels of LILRB2 and APE1 and prognosis in CRC patients

**Figure 1:**
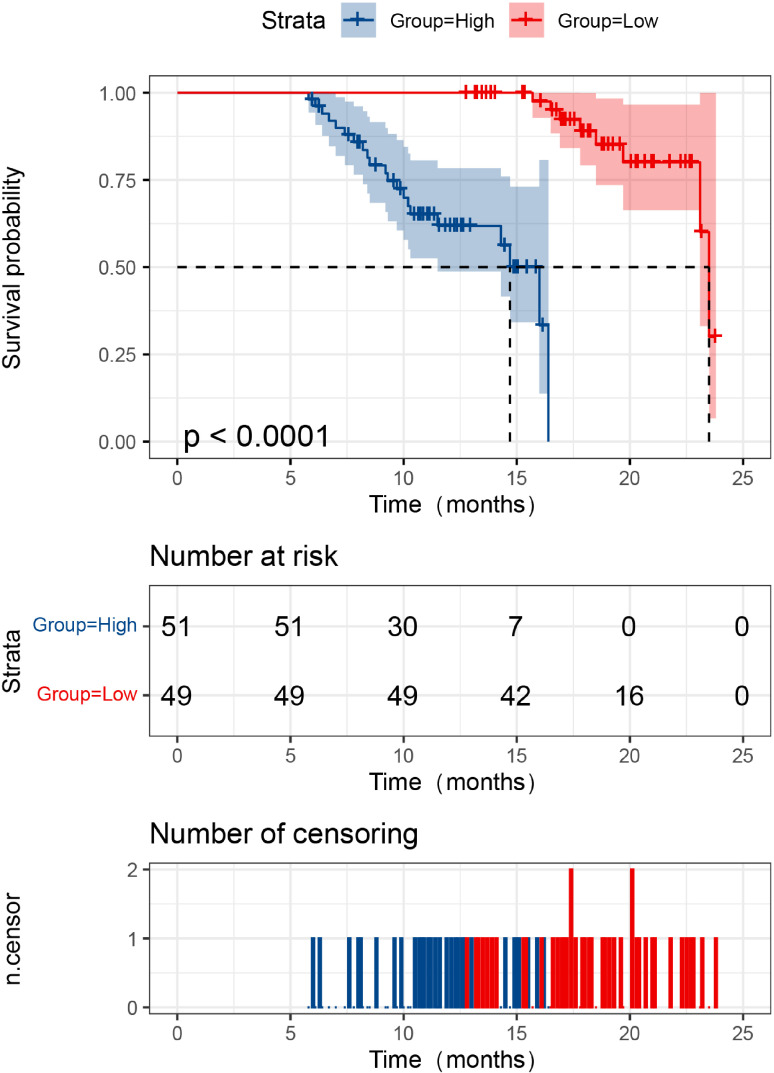
Relationship between serum LILRB2 expression level and prognosis in CRC patients. The red curve represents the survival curve of the LILRB2 low expression group, while the blue curve represents that of the high expression group. The red curve lies above the blue curve, indicating that patients in the low expression group had better survival than those in the high expression group. The *P*-value from the Log-rank test was < 0.0001, suggesting a statistically significant difference in overall survival between the two groups. The middle table indicates the number of patients still under follow-up and event-free in each group at different time points (0, 5, 10, 15, 20, and 25 months). The bottom table displays the distribution of censored events at each time point, represented by blue (high expression group) and red (low expression group) bars.

**Figure 2:**
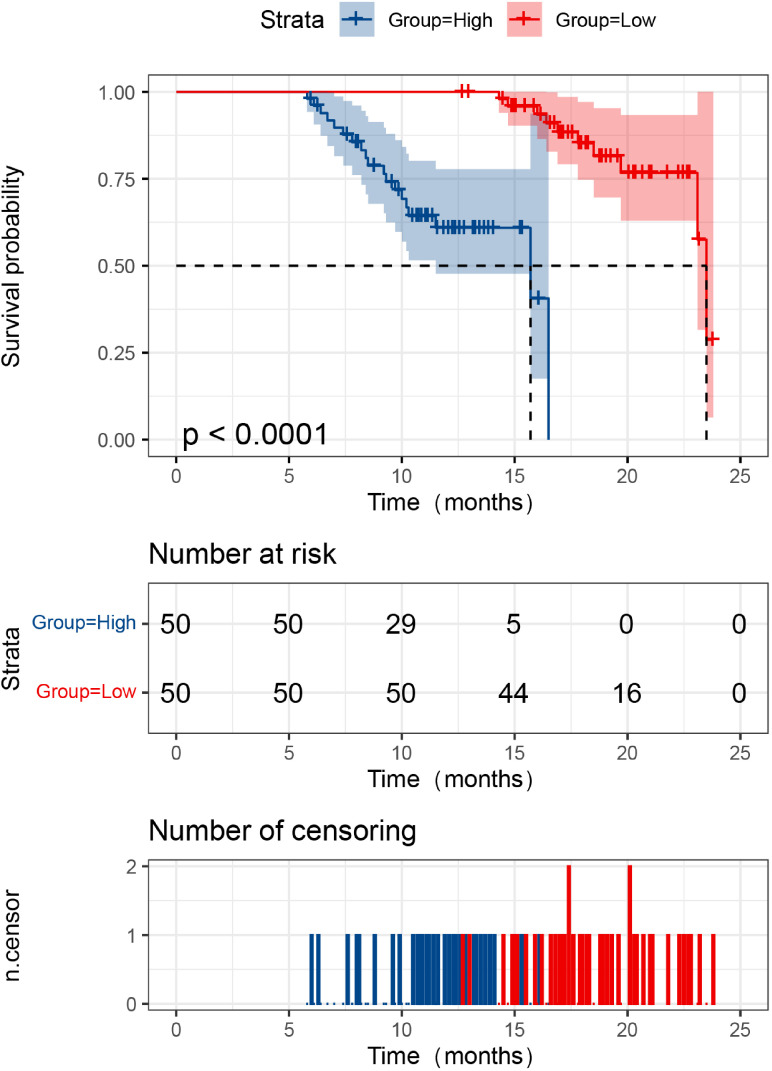
Relationship between serum APE1 expression level and prognosis in CRC patients. The red curve represents the survival curve of the APE1 low expression group, while the blue curve represents that of the high expression group. The red curve lies above the blue curve, indicating that patients in the low expression group had better survival than those in the high expression group. The *P*-value from the Log-rank test was < 0.0001, suggesting a statistically significant difference in overall survival between the two groups. The middle table indicates the number of patients still under follow-up and event-free in each group at different time points (0, 5, 10, 15, 20, and 25 months). The bottom table displays the distribution of censored events at different time points using blue (high expression group) and red (low expression group) bars.

All 100 CRC patients were successfully followed for two years, with no loss to follow-up. During this period, 29 patients died and 71 survived. The two-year survival rate in the high LILRB2 expression group was 58.82% (30/51), significantly lower than that in the low-expression group (83.67%, 41/49) (log-rank χ² = 37.284, *P* < 0.001) (**Figure 1**). Similarly, the high APE1 expression group had a survival rate of 62.00% (31/50), markedly lower than that in the low-expression group (80.00%, 40/50) (log-rank χ² = 29.960, *P* < 0.001) (**Figure 2**). These findings demonstrate that elevated serum levels of LILRB2 and APE1 are associated with poorer prognosis in CRC patients.

### Univariate and multivariate Cox analysis of prognostic factors in CRC patients

Univariate Cox regression analysis identified LILRB2 expression, APE1 expression, TNM stage, and LNM as significant factors influencing the prognosis of CRC patients (*P* < 0.05). Multivariate Cox regression further confirmed that high LILRB2 and APE1 expression levels, advanced TNM stage, and the presence of LNM were independent predictors of unfavorable prognosis (*P* < 0.05) (**Tables 3**, **4**).

**Table 3:** Assignment table.

Variable	Assignment description
Age (years)	≤ 60 = 1, > 60 = 2
Gender	Male = 1, Female = 2
Lesion location	Colon = 1, Rectum = 2
Tumor diameter (cm)	≤ 5 = 1, > 5 = 2
TNM stage	Stage I and II = 1, Stage III and IV = 2
Degree of differentiation	Moderately and well differentiated = 1, poorly differentiated = 2
Lymph node metastasis	No = 1, Yes = 2
Perineural invasion	No = 1, Yes = 2
LILRB2 expression	Low = 1, High = 2
APE1 expression	Low = 1, High = 2

**Table 4:** Univariate and multivariate Cox analysis of influencing the prognosis of CRC patients.

Variable	Univariate Cox analysis			Multivariate Cox analysis		
	HR	95% CI	*P*	HR	95% CI	*P*
Age (years)	0.496	0.228-1.081	0.078			
Gender	0.735	0.351-1.540	0.415			
Lesion location	1.016	0.475-2.172	0.968			
Tumor diameter (cm)	0.578	0.253-1.318	0.192			
TNM stage	0.148	0.054-0.405	<0.001	<0.001	<0.001-0.052	0.006
Degree of differentiation	0.629	0.238-1.661	0.350			
Lymph node metastasis	0.319	0.147-0.693	0.004	0.003	<0.001-0.326	0.015
Perineural invasion	0.728	0.350-1.511	0.394			
LILRB2 expression	0.013	0.002-0.111	<0.001	<0.001	<0.001-0.001	0.003
APE1 expression	0.050	0.014-0.177	<0.001	<0.001	<0.001-0.001	0.003

### Predictive value of LILRB2 and APE1 levels for CRC prognosis

**Figure 3:**
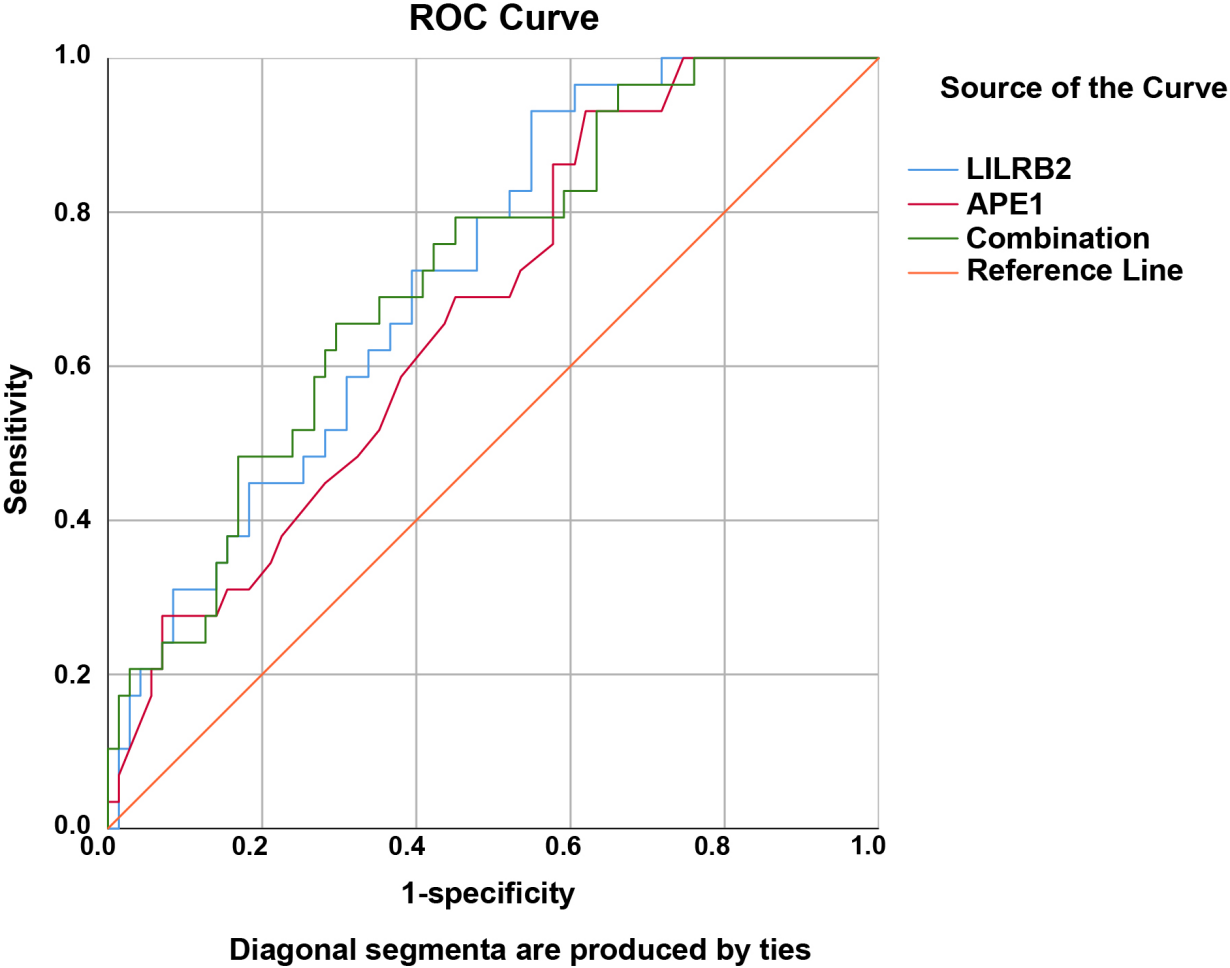
Predictive value of LILRB2 and APE1 levels for the prognosis of CRC patients as shown by ROC analysis. The AUC for LILRB2 (blue) was 0.719 (95% CI: 0.617-0.821, *P* = 0.001); for APE1 (red), 0.671 (95% CI: 0.562-0.781, *P* = 0.007); and for the combined model of LILRB2 and APE1 (green), 0.721 (95% CI: 0.616-0.826, *P* = 0.001). The orange diagonal line represents the reference line.

ROC curve analysis was used to evaluate the prognostic predictive value of LILRB2 and APE1 levels in CRC patients. The area under the curve (AUC) values for LILRB2 and APE1 were 0.719 (95% CI: 0.617-0.821, *P* = 0.001) and 0.671 (95% CI: 0.562-0.781, *P* = 0.007), respectively. The combined predictive model incorporating both markers yielded a higher AUC of 0.721 (95% CI: 0.616-0.826, *P* = 0.001), indicating improved prognostic accuracy compared with either biomarker alone (**Table 5**; **Figure 3**).

**Table 5:** Predictive value of LILRB2 and APE1 levels for the prognosis of CRC patients as shown by ROC analysis.

Test result variable (s)	Area	Std. Errora	*P*	Asymptotic 95% confidence interval	
				Lower bound	Upper bound
LILRB2	0.719	0.052	0.001	0.617	0.821
APE1	0.671	0.056	0.007	0.562	0.781
Combination	0.721	0.054	0.001	0.616	0.826

## Discussion

CRC remains one of the most prevalent malignancies of the human digestive tract. Its development follows a multistep process, beginning with benign adenomatous polyps of the large intestine and rectum that gradually transform into invasive carcinoma over time (Wu et al., 2020). Conventional treatments for CRC primarily include surgery, chemotherapy, and radiotherapy, often used in combination depending on individual patient needs. In recent years, immunotherapy has emerged as a promising therapeutic strategy for CRC (Johdi and Sukor, 2020). In this study, serum expression levels of LILRB2 and APE1 were analyzed in CRC patients to explore their correlations with clinicopathological features and prognosis. The results demonstrated that both LILRB2 and APE1 were significantly upregulated in CRC patients compared with healthy controls, suggesting that these biomarkers may play important roles in CRC onset and progression.

Specifically, LILRB2 expression was significantly associated with patient age, TNM stage, and LNM. LILRB2 is known to inhibit immune cell activity and modulate the tumor immune microenvironment through its interaction with ligands such as HLA-G, angiopoietin-like protein 2, and semaphorin-4A, thereby promoting tumor cell proliferation and metastasis (Cai et al., 2019). Consistent with earlier findings (Chen QY. et al., 2021), the current findings revealed that elevated LILRB2 levels were positively correlated with immune checkpoint proteins and LNM, contributing to tumor immune evasion and progression. Consistent with earlier evidence, overexpression of LILRB2—particularly in concert with its ligand angiopoietin-like protein 2—has been identified as a strong predictor of poor prognosis in CRC (Zhao et al., 2024). Additionally, the present results suggest that LILRB2 is relevant across different age groups, supporting its potential as a prognostic biomarker for CRC progression.

Similarly, APE1 expression was closely associated with tumor diameter, TNM stage, and LNM. Previous studies have demonstrated a strong relationship between APE1 and the tumor immune microenvironment (Li et al., 2021). APE1 expression is markedly elevated in CRC and metastatic lymph nodes compared with normal colorectal mucosa and non-metastatic lymph nodes. Its overexpression correlates with adverse pathological parameters, including lymphovascular and perineural invasion, deeper tumor infiltration, distant metastasis, and reduced overall survival (Hong et al., 2023). Consistent with these observations, our study found that patients with larger tumor diameters, advanced TNM stages, and LNM exhibited higher APE1 expression levels, further underscoring its role in CRC progression and metastasis.

Moreover, survival analysis and Cox regression models demonstrated that high expression levels of LILRB2 and APE1, advanced TNM stage, and LNM were independent risk factors for poor prognosis in CRC patients. These findings highlight the importance of incorporating these biomarkers into CRC risk stratification and therapeutic decision-making. Patients with elevated LILRB2 and APE1 expression exhibited significantly lower survival rates than those with low expression, indicating their potential as prognostic indicators for CRC outcomes. ROC analysis revealed that the combined predictive model incorporating both LILRB2 and APE1 achieved a higher AUC than either marker alone, suggesting superior prognostic accuracy when both biomarkers are evaluated in tandem. Collectively, these results suggest that LILRB2 and APE1 may serve as complementary biomarkers for assessing disease progression and predicting prognosis in CRC patients.

It is well recognized that the TNM staging system plays an irreplaceable role in determining CRC prognosis (Chen K. et al., 2021). However, as an anatomical localization-based system, it cannot fully capture the biological heterogeneity inherent to tumors (Jiang et al., 2025). In the present study, it was observed that even among patients with identical TNM stages, significant variations exist in the expression levels of LILRB2 and APE1, which could further distinguish subgroups with different survival outcomes. This finding suggests that the molecular information conveyed by LILRB2 and APE1 extends beyond the morphological boundaries defined by the TNM classification. Therefore, integrating these two molecular markers with TNM staging may enable the development of a more dynamic and predictive “anatomical-molecular” composite model for CRC prognosis. Moreover, microsatellite instability (MSI) is an important molecular phenotype in CRC, closely associated with both prognosis and responsiveness to immunotherapy (Greco et al., 2023; Jonchere et al., 2024). The present study provides new insights into the potential mechanistic links between LILRB2 and APE1 expression and MSI status in CRC. Specifically, LILRB2 may characterize a myeloid immunosuppression–dominant tumor microenvironment within microsatellite-stable (MSS) subtypes, offering a potential therapeutic target for overcoming the limited efficacy of immunotherapy in this group. Conversely, the DNA repair function of APE1 in MSI-high (MSI-H) tumors may undergo adaptive modulation, reflecting its role in maintaining genomic stability under immune pressure. Exploring the interplay between these two molecules and MSI status could significantly enhance the precision of immune-based molecular typing and the optimization of personalized treatment strategies for CRC.

Despite its novel findings, this study is not without limitations. First, it is a single-center investigation with a relatively small sample size and a follow-up period of only two years. These factors may limit the generalizability of the results and restrict the evaluation of long-term prognostic outcomes, such as the 5-year survival rate. Second, the current analysis focused solely on serum samples; validation of LILRB2 and APE1 protein expression at the tissue level through immunohistochemistry (IHC) was not performed. The lack of direct tissue-level verification somewhat weakens the evidence linking these biomarkers to the local tumor microenvironment. Most importantly, the study primarily elucidates the clinical correlations and predictive value of LILRB2 and APE1, without exploring the underlying molecular mechanisms through which they promote CRC progression.

In conclusion, the study confirms that serum levels of LILRB2 and APE1 are markedly elevated in CRC patients and are closely associated with key pathological characteristics and prognosis. These molecules represent promising biomarkers for CRC risk stratification, early diagnosis, and therapeutic planning. The novelty of this research lies in the conceptual and empirical integration of two functional molecules—originating from distinct biological pathways—into a synergistic predictive framework. This combined model demonstrated superior prognostic performance, with a higher AUC than either single indicator, thereby providing strong evidence for the refinement of CRC prognostic evaluation systems. To overcome current limitations, future studies should involve large-scale, multicenter, prospective cohorts with extended follow-up durations to validate the clinical utility of combined LILRB2 and APE1 detection. Furthermore, IHC analyses of archived tumor tissues could be conducted to verify expression localization and clinical correlations at the tissue level. Complementary *in vitro* experiments should explore the regulatory interactions and mechanistic roles of LILRB2 and APE1 in modulating the malignant phenotype of CRC cells. Ultimately, the establishment of a comprehensive prognostic prediction system that integrates clinical staging, molecular biomarkers, and molecular subtyping may enable individualized risk stratification and guide precision therapy in CRC.

## Data Availability

The raw data supporting the conclusions of this article will be made available by the authors, without undue reservation.

## References

[B1] Bray F. Ferlay J. Soerjomataram I. Siegel R. L. Torre L. A. Jemal A. (2018). Global cancer statistics 2018: GLOBOCAN estimates of incidence and mortality worldwide for 36 cancers in 185 countries. CA Cancer J. Clin. 68, 394–424. doi: 10.3322/caac.21492, PMID: 30207593

[B2] Brenner H. Kloor M. Pox C. P. (2014). Colorectal cancer. Lancet 383, 1490–1502. doi: 10.1016/S0140-6736(13)61649-9, PMID: 24225001

[B3] Cai Z. Wang L. Han Y. Gao W. Wei X. Gong R. . (2019). Immunoglobulin−like transcript 4 and human leukocyte antigen−G interaction promotes the progression of human colorectal cancer. Int. J. Oncol. 54, 1943–1954. doi: 10.3892/ijo.2019.4761, PMID: 30942436 PMC6521940

[B4] Cao M. Luan J. Zhai C. Liu H. Zhang Z. Guo N. (2025). Targeting leukocyte immunoglobulin−like receptor B2 in the tumor microenvironment: A new treatment prospect for solid tumors (Review). Oncol. Lett. 29, 181. doi: 10.3892/ol.2025.14927, PMID: 39990807 PMC11843431

[B5] Chen H. M. van der Touw W. Wang Y. S. Kang K. Mai S. Zhang J. . (2018). Blocking immunoinhibitory receptor LILRB2 reprograms tumor-associated myeloid cells and promotes antitumor immunity. J. Clin. Invest. 128, 5647–5662. doi: 10.1172/JCI97570, PMID: 30352428 PMC6264729

[B6] Chen K. Collins G. Wang H. Toh J. W. T. (2021). Pathological features and prognostication in colorectal cancer. Curr. Oncol. 28, 5356–5383. doi: 10.3390/curroncol28060447, PMID: 34940086 PMC8700531

[B7] Chen Q. Y. Chen Y. X. Han Q. Y. Zhang J. G. Zhou W. J. Zhang X. . (2021). Prognostic significance of immune checkpoints HLA-G/ILT-2/4 and PD-L1 in colorectal cancer. Front. Immunol. 12, 679090. doi: 10.3389/fimmu.2021.679090, PMID: 34054869 PMC8155601

[B8] Cubiella J. Marzo-Castillejo M. Mascort-Roca J. J. Amador-Romero F. J. Bellas-Beceiro B. Clofent-Vilaplana J. . (2018). Clinical practice guideline. Diagnosis and prevention of colorectal cancer. 2018 Update. Gastroenterol. Hepatol. 41, 585–596. doi: 10.1016/j.gastrohep.2018.07.012, PMID: 30245076

[B9] Dekker E. Tanis P. J. Vleugels J. L. A. Kasi P. M. Wallace M. B. (2019). Colorectal cancer. Lancet 394, 1467–1480. doi: 10.1016/S0140-6736(19)32319-0, PMID: 31631858

[B10] Ferkel S. A. M. Holman E. A. Sojwal R. S. Rubin S. J. S. Rogalla S. (2025). Tumor-infiltrating immune cells in colorectal cancer. Neoplasia 59, 101091. doi: 10.1016/j.neo.2024.101091, PMID: 39642846 PMC11665540

[B11] Greco L. Rubbino F. Dal Buono A. Laghi L. (2023). Microsatellite instability and immune response: from microenvironment features to therapeutic actionability-lessons from colorectal cancer. Genes (Basel) 14, 1169. doi: 10.3390/genes14061169, PMID: 37372349 PMC10298406

[B12] Hong J. Y. Oh H. H. Park S. Y. Park Y. L. Cho S. B. Joo Y. E. (2023). Expression of apurinic/apyrimidinic endonuclease 1 in colorectal cancer and its relation to tumor progression and prognosis. In Vivo 37, 2070–2077. doi: 10.21873/invivo.13304, PMID: 37652525 PMC10500501

[B13] Jiang W. Yang K. Xiao C. Ji H. Yan B. Zhao S. . (2025). Multimodal tumor microenvironment signature of colorectal cancer for prediction prognosis and chemotherapy benefit. NPJ Precis Oncol. 9, 270. doi: 10.1038/s41698-025-01069-3, PMID: 40753305 PMC12318095

[B14] Johdi N. A. Sukor N. F. (2020). Colorectal cancer immunotherapy: options and strategies. Front. Immunol. 11, 1624. doi: 10.3389/fimmu.2020.01624, PMID: 33042104 PMC7530194

[B15] Jonchere V. Montemont H. Le Scanf E. Siret A. Letourneur Q. Tubacher E. . (2024). Microsatellite instability at U2AF-binding polypyrimidic tract sites perturbs alternative sp*licing during colorectal cancer initiation*. Genome Biol. 25, 210. doi: 10.1186/s13059-024-03340-5, PMID: 39107855 PMC11304650

[B16] Li Y. Zhao X. Xiao H. Yang B. Liu J. Rao W. . (2021). APE1 may influence CD4+ naive T cells on recurrence free survival in early stage NSCLC. BMC Cancer 21, 233. doi: 10.1186/s12885-021-07950-1, PMID: 33676448 PMC7937314

[B17] Liu H. Zhang C. Peng S. Yin Y. Xu Y. Wu S. . (2025). Prognostic models of immune-related cell death and stress unveil mechanisms driving macrophage phenotypic evolution in colorectal cancer. J. Transl. Med. 23, 127. doi: 10.1186/s12967-025-06143-9, PMID: 39875913 PMC11776142

[B18] Oliveira T. T. Coutinho L. G. de Oliveira L. O. A. Timoteo A. R. S. Farias G. C. Agnez-Lima L. F. (2022). APE1/ref-1 role in inflammation and immune response. Front. Immunol. 13, 793096. doi: 10.3389/fimmu.2022.793096, PMID: 35296074 PMC8918667

[B19] Renfro L. A. Grothey A. Xue Y. Saltz L. B. Andre T. Twelves C. . (2014). ACCENT-based web calculators to predict recurrence and overall survival in stage III colon cancer. J. Natl. Cancer Inst 106, 333. doi: 10.1093/jnci/dju333, PMID: 25359867 PMC4334801

[B20] Tomasini P. P. Guecheva T. N. Leguisamo N. M. Pericart S. Brunac A. C. Hoffmann J. S. . (2021). Analyzing the opportunities to target DNA double-strand breaks repair and replicative stress responses to improve therapeutic index of colorectal cancer. Cancers (Basel) 13, 3130. doi: 10.3390/cancers13133130, PMID: 34201502 PMC8268241

[B21] Wang Q. Q. Zhou L. Qin G. Tan C. Zhou Y. C. Yao S. K. (2023). Leukocyte immunoglobulin-like receptor B2 overexpression as a promising therapeutic target and noninvasive screening biomarker for colorectal cancer. World J. Gastroenterol. 29, 5313–5326. doi: 10.3748/wjg.v29.i37.5313, PMID: 37899785 PMC10600801

[B22] Wu Z. Li Y. Zhang Y. Hu H. Wu T. Liu S. . (2020). Colorectal cancer screening methods and molecular markers for early detection. Technol. Cancer Res. Treat 19, 1533033820980426. doi: 10.1177/1533033820980426, PMID: 33353503 PMC7768867

[B23] Xue Z. Demple B. (2022). Knockout and inhibition of ape1: roles of ape1 in base excision DNA repair and modulation of gene expression. Antioxidants (Basel) 11, 1817. doi: 10.3390/antiox11091817, PMID: 36139891 PMC9495735

[B24] Zhang Z. Wu Z. Shi X. Guo D. Cheng Y. Gao J. . (2022). Research progress in human AP endonuclease 1: structure, catalytic mechanism, and inhibitors. Curr. Protein Pept. Sci. 23, 77–88. doi: 10.2174/1389203723666220406132737, PMID: 35388752

[B25] Zhao W. Z. Wang H. G. Yang X. Z. (2024). Leukocyte immunoglobulin-like receptor B2: A promising biomarker for colorectal cancer. World J. Gastroenterol. 30, 421–423. doi: 10.3748/wjg.v30.i4.421, PMID: 38313233 PMC10835539

[B26] Zygulska A. L. Pierzchalski P. (2022). Novel diagnostic biomarkers in colorectal cancer. Int. J. Mol. Sci. 23, 852. doi: 10.3390/ijms23020852, PMID: 35055034 PMC8776048

